# Clinical Features of Acute Rhabdomyolysis in 55 Pediatric Patients

**DOI:** 10.3389/fped.2020.00539

**Published:** 2020-09-04

**Authors:** Zhengxiong Yao, Ping Yuan, Siqi Hong, Mei Li, Li Jiang

**Affiliations:** Department of Neurology, Chongqing Key Laboratory of Translational Medical Research in Cognitive Development and Learning and Memory Disorders, Ministry of Education Key Laboratory of Child Development and Disorders, National Clinical Research Center for Child Health and Disorders, China International Science and Technology Cooperation Base of Child Development and Critical Disorders, Children's Hospital of Chongqing Medical University, Chongqing, China

**Keywords:** rhabdomyolysis, children, etiology, neuromuscular disease, immunoencephalitis

## Abstract

**Objective:** This study was designed to compare the clinical manifestations, laboratory tests, etiology, and prognosis of children with acute rhabdomyolysis (RM) at various ages. This study was designed to analyze the risk factors for acute kidney injury (AKI) in children with RM and to identify the role of neuromuscular and autoimmune disease in children with RM.

**Methods:** Clinical data for 55 children with RM were collected and statistically analyzed. Patients were stratified to an infant group (G1) (age <1 year), preschool group (G2) (age 1–6 year), school-age group (G3) (age 7–11 year), and an adolescent group (G4) (age 12–16 year).

**Results:** The top three clinical manifestations were dark urine (52.7%), myalgia (38.2%), and fever (23.8%). Patients in G1 had fever (71.4%), vomiting (77.8%), and urinalysis abnormalities (14.3%), without triad clinical manifestations. Fifty percent of patients in G4 group had myalgia; 70.8% had dark urine; 75% had abnormal urine tests. The most common cause in each age group was as follows: sepsis (57.1%) in G1; hereditary neuromuscular diseases (44.4%) in G2; immune diseases (40%) in G3; strenuous exercise (50%) in G4. Logistic regression analysis shown that AKI was not corelated with age, gender, or peak creatine phosphokinase. AKI was, however, associated with presence of an electrolyte disorder.

**Conclusion:** The clinical manifestations and laboratory findings in infants with acute RM are not typical and need to be taken seriously. The presence of an electrolyte disorder is a risk factor for AKI in children with RM. The most common pathogenesis of RM varies among age groups. Congenital hereditary metabolic disease and immune diseases should not be ignored as a cause of RM in children.

## Introduction

Rhabdomyolysis (RM) is a group of clinical syndromes that cause skeletal muscle damage. A lapse in the integrity of the cellular membrane allows a large amount of cellular contents (such as enzymes, proteins, ions, etc.), especially myoglobin, to rapidly enter the circulation and urine. Typical manifestations of RM are myalgia and muscle weakness with dark urine (1). However, the occurrence of RM in children is easily ignored clinically due to atypical clinical manifestations, resulting in serious complications including acute renal failure (2). With the development of detection technology in recent years, the etiology spectrum of RM in children has been greatly expanded. More than 60 single-gene genetic diseases have been found to associate with RM (3). In addition, more and more immune diseases have been identified as important causes of RM (4, 5).

Because the bodies of infants are in various stages of organ development and functional maturity, they respond differently to the large number of cytolysates in blood and urine, making it difficult to unify the clinical characteristics of RM. To better understand the characteristics of RM in children at different ages, in order to improve diagnosis and treatment, we compared clinical manifestations, laboratory tests, etiology, and prognosis. We also focused on the etiology of hereditary neuromuscular and autoimmune diseases and risk factors for acute kidney injury (AKI).

## Materials and Methods

### Patients and Groups

This study was a retrospective single-center medical chart review of patients with a diagnosis of RM. The patients were diagnosed by their medical histories and elevated serum creatine phosphokinase (CK) levels (>1,000 IU/L), then admitted to the Children's Hospital of Chongqing Medical University from January 2010 to August 2019. Patients were excluded if they had: (1) patients of muscle disorders without any clinical symptoms of RM; (2) a history of myocardial damage with a documented creatine kinase isoenzyme (CK-MB) fraction above 5%. The clinical data for 55 children with RM were collected. A standardized form was applied to review the medical records. All charts were reviewed by at least two authors. This project was authorized by the Ethics Committee of Chongqing Medical University. Written informed consent was obtained from participants.

Cases were divided into four groups according to the patient's age at the time of the visit: (1) infant group (G1) (age <1 year); (2) preschool group (G2) (age 1–6 year); (3) school-age group (G3) (age 7–11 year); (4) adolescent group (G4) (age 12–16 year).

### Data Collection

Information on medical history and laboratory tests was collected. The medical history information included age, gender, complaints, symptoms of infection (fever, cough, nasal obstruction, sore throat, vomiting, diarrhea, etc.), myalgia, muscle weakness, dark urine, oliguria, convulsions or disturbance of consciousness, multiple-organ dysfunction, history of exercise or trauma, past history (developmental milestones and history of RM), treatment plan, and outcome. The laboratory data analyzed included initial CK, peak CK, CK-MB, myoglobin (Mb), lactate dehydrogenase (LDH), aspartate aminotransferase (AST), alanine aminotransferase (ALT), urea nitrogen, blood electrolytes, serum creatinine, and urine routine. Cause classification was based on the patient's discharge diagnosis or follow-up assessment by specialists.

### Statistical Analysis

Statistical processing was performed using SPSS 17.0 software (IBM-SPSS; Chicago, IL, USA). The count data are expressed as percentages; the measurement data (such as CK, ALT, AST, CK-MB, and LDH) have been converted to natural logarithms and are expressed as mean ± standard deviation (x ± s). Statistical comparisons were performed using the chi-square test or one-way ANOVA, and logistic regression was used to analyze risk factors for AKI. *P* < 0.05 was considered as a significant difference.

## Results

### Clinical Manifestations

Fifty-five patients with RM (37 males and 18 females) were included in this study. Median age was 11 yr (inter-quartile range: 5.5–11 year). Twenty-three cases (41.8%) had fever; 13 cases (23.6%) had vomiting; 21 cases (38.2%) had myalgia; 15 cases (27.3%) had muscle weakness; 29 cases (52.7%) had dark urine; 16 cases (29.1%) had convulsions and/or consciousness disorder (Table 1). Significant differences were found among the groups in the clinical manifestations of fever, vomiting, and dark urine. Six cases (77.8%) in the infant (G1) group were admitted because of vomiting. No patients with myalgia, muscle weakness, or dark urine were found in G1, suggesting that the clinical manifestations of RM in infants were not typical. This may be a reason for the condition to be misdiagnosed as digestive tract infection or intracranial infection. However, in the adolescent (G4) group, 12 cases (50%) had myalgia; 8 cases (33.3%) had muscle weakness; 17 cases (70.8%) had dark urine. These findings indicate that elder children have more typical clinical manifestations (Table 1).

**Table 1:** Classification of clinical manifestations of RM among groups.

	**Total**	**G1**	**G2**	**G3**	**G4**	***P***
*N*	55	7	9	15	24	
Gender (M:F)	37:18	3:4	6:3	9:6	19:5	0.278
Fever *n* (%)	23 (41.8)	5 (71.4)	7 (85.7)	7 (46.7)	4 (16.7)	0.002
Vomiting n (%)	13 (23.6)	6 (77.8)	2 (22.2)	2 (13.3)	3 (12.5)	0.001
Myalgia *n* (%)	21 (38.2)	0	3 (33.3)	6 (40.0)	12 (50.0)	0.106
Muscle weakness *n* (%)	15 (27.3)	0	4 (57.1)	3 (20.0)	8 (33.3)	0.198
Dark urine *n* (%)	29 (52.7)	0	6 (66.7)	6 (40.0)	17 (70.8)	0.004
C/C *n* (%)	16 (29.1)	4 (57.1)	3 (33.3)	5 (33.3)	4 (16.7)	0.191

*G1, age <1 year; G2, age 1–6 year; G3, age 7–11 year; G4, age 12–16 year; N, number of patients; M, male; F, female; C/C, convulsions and/or consciousness disorder*.

### Etiology

The top four causes in the RM patient groups were exercise (23.6%), infection (16.4%), immunity (16.4%), and myopathy (14.5%), respectively. The most common etiologies among age groups were as follows: sepsis (57.1%) in the infant (G1) group; hereditary neuromuscular disease (44.4%) in the preschool (G2) group; immune disease (40%) in the school-age (G3) group; strenuous exercise (50%) in the adolescent (G4) group (Table 2).

**Table 2:** Etiology of RM among groups.

	**G1**	**G2**	**G3**	**G4**	**Total *n* (%)**
*N*	7	9	15	24	55
Infection	4	1	4	0	9 (16.4)
Exercise	0	0	1	12	13 (23.6)
Immunology	0	2	6	1	9 (16.4)
Genetic	0	4	2	2	8 (14.5)
Metabolism	1	0	0	1	2 (3.6)
Poison/drug	0	1	0	3	4 (7.2)
Trauma	1	0	2	1	4 (7.2)
Unknown	1	1	0	4	6 (10.9)

*N, number of patients*.

Notably, eight patients (14.5%) were diagnosed with hereditary neuromuscular disease (Table 3). Four cases were admitted with RM at the time of their first visit to the hospital. In three cases previously diagnosed with hereditary neuromuscular disease, RM was induced by either infection or strenuous exercise. One case was a 22-mo-old male with RM who was admitted because of vomiting and diarrhea. He had language development retardation, occasional walking instability, and mildly impaired coordination. His siblings died of vomiting, diarrhea, and coma at 3 years of age. His muscle biopsy did not show any specific changes. However, whole-exon sequencing revealed a heterozygous mutation in the *TANGO2* gene [c.706A>G(p.T236A), c.365T>A(p.I122K)]. The patient was therefore diagnosed with *TANGO2* variant-related metabolic encephalopathy with RM (Patient 7 in Table 3).

**Table 3:** Data for RM patients with hereditary myopathy.

	**Gender**	**Age (month)**	**trigger**	**Clinical manifestation**	**Initial CK (IU/L)**	**Peak CK (IU/L)**	**Urine routine (dipstick)**	**Diagnosis**
1	M	19	Infection	Fever, Muscle weakness	9,822	21,659	Normal	MB: DMD
2	M	60	Infection	Fever, vomiting, myalgia, muscle weakness, dark urine	9,531	15,682	Protein3+, heme3+	Gene DMD
3	M	138	NA	Vomiting	1,265	13,151	Protein1+, heme 3+	MB: DMD
4	M	84	Infection	Fever, myalgia, dark urine	NA	83,402	Protein3+, heme3+	Gene DMD
5	M	156	Exercise	Myalgia, dark urine	5,802	29,606	heme1+	Gene DMD
6	M	21	Infection	Fever, myalgia, dark urine, oliguria	3,784	24,998	Protein 1+	Gene *POMT2*
7	M	24	Infection	Vomiting, muscle weakness, dark urine, oliguria	1,069	12,6994	Protein2+, heme3+	Gene *TANGO2*
8	F	168	Exercise	Muscle weakness, dark urine	2,334	69,715	Protein3+, heme3+	MB: mitochondrial myopathy

*M, male; F, female; NA, not available; MB, muscle biopsy; CK, creatine phosphokinase; DMD, Duchenne muscular dystrophy*.

In addition, nine patients (16.4%) in this study had autoimmune diseases (Table 2). Among them, four cases had autoimmune encephalitis with RM during treatment. Two of them were anti-N-methyl-D-aspartate receptor (NMDAR) encephalitis(the titer in cerebrospinal fluid were 1:100 and 1:,320 respectively); one was voltage-gated potassium channel complex (VGKC) antibody-mediated encephalitis; another case was anti-NMDA antibody combined with myelin oligodendrocyte glycoprotein (MOG) antibody encephalitis. Dark urine appeared after treatment with gamma globulin and/or glucocorticoids in these four cases. Vomiting and myalgia with muscle weakness happened in one patient with neuromyelitis optica after long-term use of prednisone.

### Laboratory Tests

No significant differences between groups were found in CK, CK-MB, Mb, LDH, ALT, or AST (Table 4), indicating that there was no age-related difference in levels of these markers among children with RM. However, urine abnormalities differed significantly among groups (*P* = 0.039); urine abnormalities (hematuria or proteinuria) were significantly less common in the infant (G1) group (14.3%), compared with the other groups. Twenty patients had electrolyte disorders, including 17 cases with hypokalemia and 12 cases with hypocalcemia; however, there was no significant difference among groups (*p* = 0.863). Twenty-seven patients (49.1%) had AKI; nevertheless, the incident rate of AKI was not different among the groups.

**Table 4:** Laboratory results for all groups.

	**G1**	**G2**	**G3**	**G4**	***P***
*N*	7	9	15	24	
CK (IU/L)	1,677.1	60,475.3	64,130.2	39,622.3	0.271
Mb (IU/L)	3,744.0	5,052.0	5,175.0	5,998.0	0.893
CK-MB (U/L)	280.8	568.1	246.7	277.0	0.270
LDH (U/L)	2,967.2	4,244.2	4,396.8	3,434	0.743
ALT (U/L)	921.7	891.6	657.8	374.6	0.341
AST (U/L)	1,200.0	1,766.0	1,751.0	1,369.0	0.696
Electrolyte disorder n (%)	2 (28.6)	3 (33.3)	7 (46.7)	8 (33.3)	0.863
Abnormal urine routine n (%)	1 (14.3)	6 (66.6)	10 (66.7)	18 (75.0)	0.039
AKI n (%)	3 (42.9)	4 (44.4))	8 (53.3)	12 (50.0)	1.000

*N, number of patients; CK, creatine phosphokinase; CK-MB, creatine kinase isoenzymes; LDH, lactate dehydrogenase; ALT, alanine aminotransferase; AST, aminotransferase; AKI, acute kidney injury*.

To explore the factors involved in AKI, logistic regression was then applied to analyze the relationship between AKI and patient gender, age, peak CK, and presence of an electrolyte disorder (Table 5). The results indicated that AKI had no relationship with gender, age, or peak CK levels, but AKI was significantly associated with the presence of an electrolyte disorder. Eighteen of the 27 patients (66.7%) with AKI had an electrolyte disorder, while only two of 28 patients without AKI had an electrolyte disorder, suggesting that the presence of an electrolyte disorder was a risk factor for AKI among patients with RM.

**Table 5:** Logistic regression analysis for risk factors for AKI in children with RM.

	**OR (95%CI)**	***P***
Gender	8.675 (0.567–132.681)	0.121
Age	0.899 (0.700–1.142)	0.382
CK	1.154 (0.975–1.390)	0.133
Electrolyte disorder	92.526 (5.704–1,500.862)	0.001

*OR, odds ratio; CK, creatine phosphokinase*.

### Outcomes

In the G1 group, an infant with very long-chain acyl-CoA dehydrogenase deficiency died after 8 days in the intensive care unit. The remaining six patients improved after hydration, alkalinization, and/or renal replacement therapy (RRT). Two cases in the G2 group were diagnosed with severe sepsis, and FIRES were discharged abnormally; these patients died shortly afterwards. All of the remaining seven cases in G2 were discharged with recovery. One case in G3 received RRT, and three cases (diagnosed as anti-NMDAR encephalitis, sepsis, and car accident, respectively) died shortly after abnormal discharge. The remaining 11 cases were fully recovered. All cases in G4 recovered after discharge, and 11 were treated with RRT. The condition of the child with RM combined with *TANGO2* variant-related metabolic encephalopathy improved after treatment with hydration and alkalinization; this patient's CK levels returned to normal at the time of discharge. We adjusted the patient's mealtime and increased the amount of raw corn starch in his diet. No RM recurrence was observed at the 1-year follow-up visit. All patients were followed by telephone, and 41 were successfully contacted. No patient had a recurrence of RM. There was no difference in outcomes among the four groups.

## Discussion

RM is typically characterized by a clinical triad: myalgia, muscle weakness, and dark urine. However, <10% of children with RM in this study had such typical signs. Indeed, more than 50% of patients only have dark urine without myalgia or muscle weakness (6, 7)^.^ The most common manifestation in this study was dark urine (52.7%), followed by fever (41.8%). Our data indicate that the clinical manifestations in children with RM are age related, which is similar to Chen's findings (8). The younger the age is, the more atypical are the clinical manifestations. In the infant (G1) group, the common clinical manifestations were fever and vomiting with convulsions or disturbance of consciousness. The infants were not able to describe their feelings of myalgia and had negative urine test results; they did not have the typical triad of clinical manifestations. In this condition, they are easily misdiagnosed with other diseases such as encephalitis. The differential diagnosis requires careful attention. However, for older children with RM, especially adolescents (G4), the clinical triad was more common, and the incidence of positive urine test results was higher.

AKI is a common serious complication of RM that may be induced by myoglobinuria, hypovolemia, and/or metabolic acidosis, the probability of which is 10–60% (9). Studies have reported that the factors affecting AKI in patients with RM are serum concentrations of potassium, creatinine, and albumin (10). Other studies have shown that serum calcium, phosphorus, potassium, and uric acid levels are independent predictors of AKI (11). A high CK level has been reported as a risk factor for AKI (12): when CK peak is >5,000 U/L, the incidence of AKI is about 19%; however, when the CK level is <5,000 U/L, the incidence of AKI is only 8%. However, the correlation of CK peak level with AKI development has not previously been reported (13). Our data showed that the overall incidence rate of AKI among patients with RM was 49.1%. No significant difference between age groups was found in peak CK or Mb. Complications of AKI were not found to correlate with age, gender, or peak CK levels; however, complications of AKI were associated with the presence of an electrolyte disorder.

RM can result from various disorders including trauma, infection, excessive exercise, inflammation, drugs, metabolism, and heredity (14). It has previously been reported that the most common causes of adult RM are trauma and drugs (up to 80% of cases); however, the top pathogens in pediatric RM are infections and congenital diseases (15, 16). In this study, the most common cause of pediatric RM was strenuous exercise (23.6%), followed by infection (16.4%) and autoimmune diseases (16.4%). The most common cause of RM among babies is infection. Nevertheless, excessive exercise is an important cause among children with RM, especially adolescents. Studies have reported that pre-exercise factors, such as poor physical condition, high humidity, restrictive clothing, use of anticholinergics, hypokalemia caused by excessive sweating, and the use of drugs that enhance strength, may be associated with RM after exercise (17). In addition, muscle tension (such as running downhill) causes eccentric contractions, which are more likely to induce muscle injury than concentric contractions (18).

With the development of genetic testing, more hereditary causes including metabolic myopathies, structural myopathies, channel-related gene mutations, and other congenital hereditary metabolic diseases have been found to be associated with RM. Exome sequencing has revealed that 43% of RM cases have single-gene mutations (19). It has been reported that the most common neuromuscular disease that causes RM is Fukutin-related protein muscular dystrophy; the next most common pathogens are anoctaminopathy-5, calpainopathy-3, and dystrophinopathy (20). Moreover, dysferlinopathy, dystrophinopathy, and caveolinopathy-3 can manifest with RM in female carriers. In particular, 36% of limb-girdle muscular dystrophy 2I (LMGD2I) cases may experience recurrent RM (9). Chan also reported an increased risk of RM in patients with some hereditary neuromuscular diseases, such as Duchenne muscular dystrophy (DMD)/Becker muscular dystrophy (BMD), fukutin-related protein, dysferlin, γ-sarcoglycans, and anoctamin 5 deficiency-associated muscular atrophy (21). In this study, the most common hereditary neuromuscular disease associated with RM was DMD, which may be unique to this Chinese population. It should be noted that some patients with myopathy have no clinical manifestations during the interval, and acute RM may be induced by certain triggers, such as strenuous activity, fasting, fever, infection, cold exposure, anesthesia, and drugs (20). The presence of hereditary metabolic myopathy should be investigated in any RM patient with any of the following conditions: recurrent RM, exercise intolerance, ptosis, eye muscle paralysis, dystonia, exercise-induced muscle spasm or muscle weakness, dark urine and myalgia induced by minimal exercise, family history of myopathy (3). There was a *TANGO2* variant-related metabolic encephalopathy with RM in this study. The patient's clinical features were consistent with other reports (22). The disease manifested as recurrent acute metabolic crisis (hypoglycemia, hyperlactosis, mild hyperammonemia), muscle weakness, and RM, accompanied by ataxia, disorientation, coma, and other acute encephalopathy. The patient may eventually die from recurrent ventricular tachycardia and torsades de pointes. Mental retardation, poor coordination, seizures, and hypothyroidism may happen (23).

RM could also happen in patients with immune encephalitis (24, 25). Nine patients included in the study had autoimmune diseases, three of whom had anti-NMDAR encephalitis. They usually had seizures, dystonia, and increased involuntary movement. An important hypothesis is that the recovery of NMDA receptors after immunotherapy can suppress the dopaminergic system, leading to hypersensitivity to dopamine receptor blockers, which is likely to cause RM and neuroleptic malignant syndrome (24). However, in our study, only one case with VGKC antibody-mediated encephalitis was treated with chlorpromazine, haloperidol, and risperidone in succession. One patient with anti NMDAR encephalitis was treated with haloperidol. Another two patients with anti-NMDAR encephalitis were never treated with a dopamine receptor blocker. The reason for their RM could not be explained by this hypothesis. Some immune diseases such as systemic lupus erythematosus are reported as an important cause of RM (4). We suspect that RM may be related to the special immune status in those patients. Notably, one study reported treatment with short-term high-dose steroid therapy in one RM patient who could not take intravenous fluids (26). The specific pathogenesis is still unclear. More cases and further research are needed urgently.

**Figure 1:**
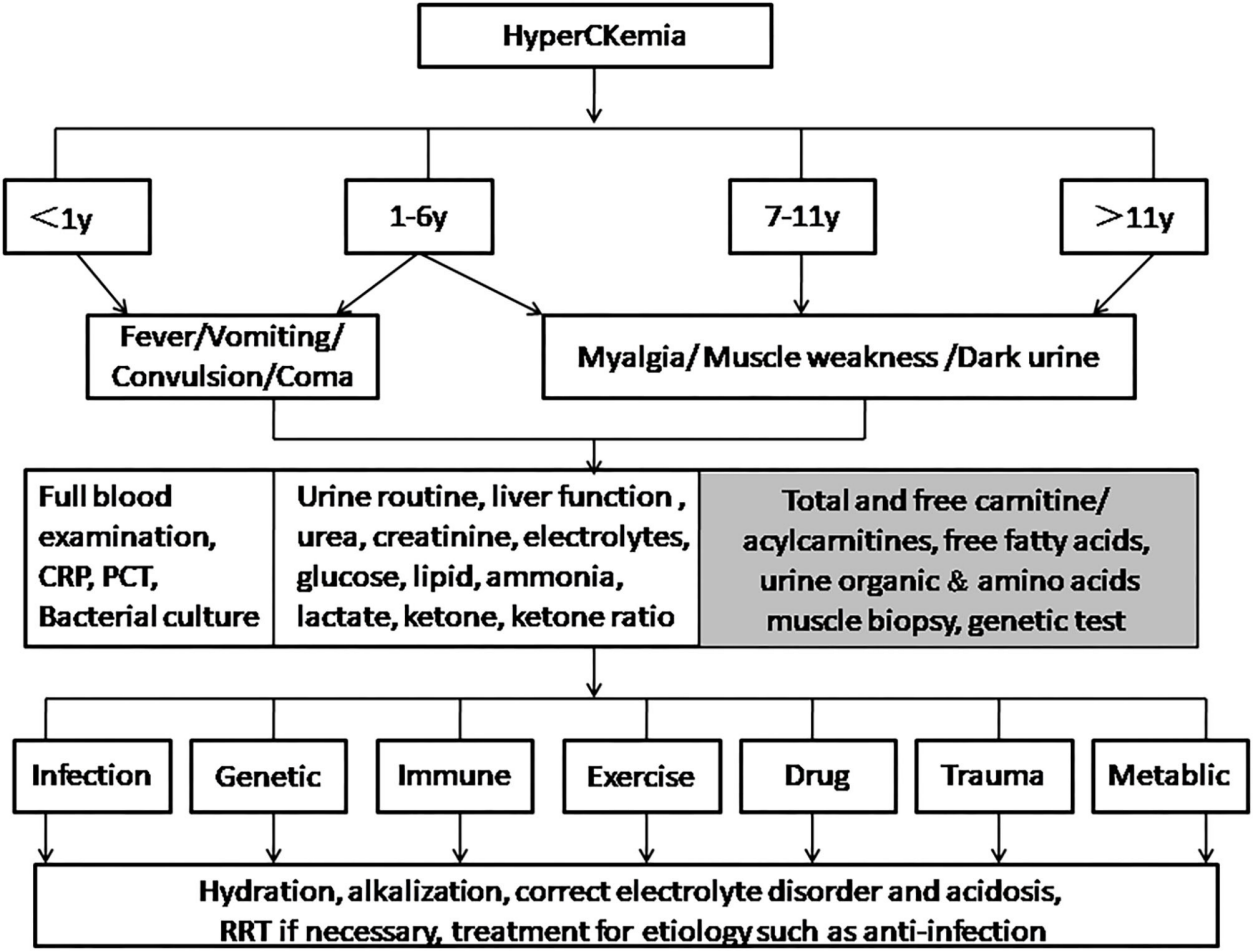
Management of hyperCKemia in pediatric patients. For children younger than 6 years old, especially infants, it is necessary to pay attention to atypical symptoms such as fever, vomiting, convulsions, and unconsciousness. Tests such as full blood test and bacterial culture are required to determine whether there is infection causes. Children older than 6 years old are more prone to the typical triad. Tests in second box are routine. If there are abnormalities in glucose, lipids, ammonia, lactate, or ketone, items in the gray box are optionally done. Clinicians should try their best to distinguish the etiology to take appropriate treatments.

We made a proposal of a flow-chart related to the management of hyperCKemia in pediatric patients (Figure 1). We expected it to help improve the diagnosis and treatment of RM for pediatric clinicians.

## Conclusion

The clinical features of 55 children with RM were analyzed in different age groups. The results indicated that atypical manifestations in younger children usually obscure the diagnosis and complicate treatment. Electrolyte disorder was a risk factor for AKI in the children with RM. In contrast to the etiology observed in adult cases, the most common cause of RM in children was strenuous exercise, followed by infection and immune disorders. Also, congenital hereditary metabolic disease should not be ignored as a cause of RM in children.

## Data Availability Statement

All datasets generated for this study are included in the article/supplementary material.

## Ethical Statement

This project was authorized by the Ethics Committee of Chongqing Medical University. Written informed consent was obtained from the parents.

## Author Contributions

PY designed the study and wrote the manuscript. ZY and PY collected and analyzed data. SH, ML, and LJ helped to analyze the data. All authors contributed to the article and approved the submitted version.

## Conflict of Interest

The authors declare that the research was conducted in the absence of any commercial or financial relationships that could be construed as a potential conflict of interest.

## Abbreviations

RM: rhabdomyolysis
AKI: acute kidney injury
ALT: alanine aminotransferase
AST: aminotransferase
CK: creatine phosphokinase
CK-MB: creatine kinase isoenzymes
Mb: myoglobin
DMD: Duchenne muscular dystrophy
BMD: Becker muscular dystrophy
FIRES: febrile infection-related epilepsy syndrome
LDH: lactate dehydrogenase
NMDAR: N-methyl-D-aspartate receptor
VGKC: voltage-gated potassium channel complex
RRT: renal replacement therapy.
